# Ethnopharmacological and Chemical Characterization of *Salvia* Species Used in Valencian Traditional Herbal Preparations

**DOI:** 10.3389/fphar.2017.00467

**Published:** 2017-07-25

**Authors:** Vanessa Martínez-Francés, Emeline Hahn, Segundo Ríos, Diego Rivera, Eike Reich, Roser Vila, Salvador Cañigueral

**Affiliations:** ^1^Estación Biológica-Jardín Botánico Torretes, Instituto de la Biodiversidad, Centro Iberoamericano de la Biodiversidad, Universidad de Alicante Alicante, Spain; ^2^Unitat de Farmacologia, Farmacognòsia i Terapèutica, Facultat de Farmàcia i Ciències de l'Alimentació, Universitat de Barcelona Barcelona, Spain; ^3^Departamento de Biología Vegetal, Campus de Espinardo, Universidad de Murcia Murcia, Spain; ^4^CAMAG Laboratory Muttenz, Switzerland

**Keywords:** *Salvia blancoana* subsp. *mariolensis*, *Salvia officinalis* subsp. *lavandulifolia*, *Salvia x hegelmaieri*, ethnopharmacology, essential oil, GC-MS, HPTLC, quality control

## Abstract

In Valencia Region (Spain), some wild and cultivated sages are used for medicinal purposes. Among them, *Salvia officinalis* subsp. *lavandulifolia* (SL) is widely employed and known for production of Spanish sage oil and herbal products. Nevertheless, it shares the market with *S. blancoana* subsp. *mariolensis* (SB) and, to a lesser extent, with their hybrid *S*. x *hegelmaieri* (SH). The knowledge on these two species is far low and confusion between them is possible. The aim of the present paper is to improve the ethnopharmacological, morphological and chemical knowledge of these sages, and to contribute to setting up quality specifications for improving identification and distinction from other *Salvia* species, such as, *S. officinalis* subsp. *officinalis, S*. x *auriculata* and *S. microphylla* var. *microphylla*. Samples were collected in Valencia Region and surrounding mountain areas during the ethnopharmacological field work. Twenty-nine medicinal uses were reported for SL, 13 of them being also recorded for SB. Of particular interest is a homemade liquor, used as digestive and known as “salvieta,” which is mainly prepared with SB. The macro- and microscopic characters are insufficient for identification of cut, crushed or powdered material. The study of the essential oil and a HPTLC (High Performance Thin Layer Chromatography) fingerprint of their extracts could help to distinguish SB from the other sages. The essential oil from dried aerial parts of SB (content: 1.8–4.5%) was characterized by GC-FID (Gas Chromatography with Flame Ionization Detector) and GC-MS (Gas Chromatography coupled to Mass Spectrometry) showing a composition close to that currently accepted for Spanish sage essential oil in the European Pharmacopoeia, ISO (International Standard Organization) and UNE (Una Norma Española) standards, with 1,8-cineole (13.7–45.7%) and camphor (12.1–28.6%) as major constituents. HPTLC methods, based on the analysis of hydroalcoholic and dichloromethane extracts, allowed to distinguish SB from other *Salvia* taxa currently found in Valencia region, except from its hybrid SH. This interdisciplinary study, that combines popular knowledge with botany and chemistry, allows to identify the raw herbal material from SB and to distinguish it from other *Salvia* species, ensuring a proper commercialization as herbal teas or for the preparation of spirits.

## Introduction

The promotion of European plant derived products (particularly those coming from rural areas), such as, wines and spirits, with specific characteristics determined by the geographical origin, including the area of production, has experienced a significant increase, especially in the last decade (Qin et al., 2012). This reflects the large popularity of these local products, which particularly in Spain benefit from a great acceptance. Some examples of Protected Geographical Indications (PGI) include liquors, such as, “Cantueso alicantino,” “Palo de Mallorca” or “Ratafía catalana”; anise-flavored spirits like “Anís Paloma Monforte del Cid,” “Hierbas Ibicencas” and “Hierbas de Mallorca”; or other alcoholic drinks, such as, “Herbero de la Sierra Mariola,” “Aperitivo Café de Alcoy,” “Aguardiente de hierbas de Galicia,” and “Pacharán de Navarra” (E-Spirits-Drinks, 2017). Many of them are made in the Eastern part of the Iberian Peninsula or in the Balearic Islands as an evolution of traditional herbal preparations, which nowadays have a social use as drinks in family gatherings and festivals.

Most of the ingredients in these preparations are aromatic plants that have been used in traditional medicine for centuries (Liang et al., 2004; Bouaziz et al., 2009). The majority are Lamiaceae, such as, *Salvia* species, intensively studied because of their economic interest for food, cosmetic, and pharmaceutical preparations (Durling et al., 2007; Longaray Delamare et al., 2007; Walch et al., 2011; Orhan et al., 2012). In particular, *S. officinalis* L. subsp. *officinalis* and S. *officinalis* L. subsp. *lavandulifolia* (Vahl) Gams (syn. *S. lavandulifolia* Vahl) have been largely investigated due to their biological activities as anti-nociceptive, anti-inflammatory, antioxidant and inhibitor of cholinesterase activity (Bakkali et al., 2008; Adorjan and Buchbauer, 2010; Ramos et al., 2010; Kennedy and Wightman, 2011; Walch et al., 2011). In addition, S. *officinalis* subsp. *lavandulifolia* is the accepted source for the Spanish sage oil in the European Pharmacopoeia (EDQM, 2016) and other standards (AENOR, 2001; ISO, 2005).

In Valencian region (Spain), several wild and cultivated *Salvia* species are traditionally used in folk medicine as herbal tea or for digestive liquors (Martínez-Francés and Ríos, 2005). Among them, *S. blancoana* Webb. Heldr. subsp. *mariolensis* Figuerola, an Iberolevantine endemism, S. *officinalis* subsp. *lavandulifolia* and their hybrid *S. x hegelmaieri* Porta Rigo, are increasingly found in local markets, especially in the Southern part of Valencia region (Martínez-Francés et al., 2012). Thus, quality control of the herbal raw material is important for a proper commercialization of these species. Identification using morphological and/or chemical markers is an essential step of this control.

Taxonomy of Iberian *Salvia* species related to *S. officinalis*, such as, *S. blancoana*, and the previously known as *S. lavandulifolia*, is controversial regarding their status and level (Alcaraz and De la Torre, 1988; Alcaraz et al., 1988; Bolòs and Vigo, 1995; Laguna et al., 1998; López, 2001; Reales et al., 2004; Serra, 2007; Sáez, 2010; Mateo and Crespo, 2014). In the case of *S. blancoana*, a consensus exist of its status as species, including several subspecies, among them *S. blancoana* subsp. *mariolensis* (Laguna et al., 1998; Reales et al., 2004; Serra, 2007; Mateo and Crespo, 2014) which is mainly distributed in the Alcoy—Denia eastern mountains, reaching the Ayora—Villena western mountains, a region between North of Alicante province and South of Valencia province (Serra, 2007).

Essential oil composition of Iberian sages has been extensively studied (Marcos Sanz et al., 1988; Guillén and Manzanos, 1999; Jordán et al., 2009; Herraiz-Peñalver et al., 2010; Ramos et al., 2010; Martínez-Francés et al., 2012; Santana et al., 2012). However, an exhaustive chemical characterization of the oil of *S. blancoana* subsp. *mariolensis* an *S. x hegelmaieri* is lacking.

There is a need of a better morphological and chemical knowledge of *S. blancoana* subsp. *mariolensis*, which could help the establishment of quality specifications for the herbal materials and improve the opportunities of marketing the herbal preparations thereof such as, teas and traditional liquors under controlled conditions. For this reason, the aims of the present paper were: to analyze the ethnopharmacological knowledge reported about *S. blancoana* subsp. *mariolensis* in comparison with S. *officinalis* subsp. *lavandulifolia*; to clarify the main morphological characters that can help the identification and differentiation of the sages marketed in the studied area; to characterize the essential oil of *S. blancoana* subsp. *mariolensis* by GC-FID (Gas Chromatography with Flame Ionization Detector) and GC-MS (Gas Chromatography coupled to Mass Spectrometry) and comparing its composition with those of closely related taxa; and to develop a method of identification by HPTLC (High Performance Thin Layer Chromatography) to distinguish *S. blancoana* subsp. *mariolensis* from other *Salvia* taxa currently found in Valencia region, that could be routinely applied for quality control of herbal material.

## Materials and methods

### Study area

**Figure 1:**
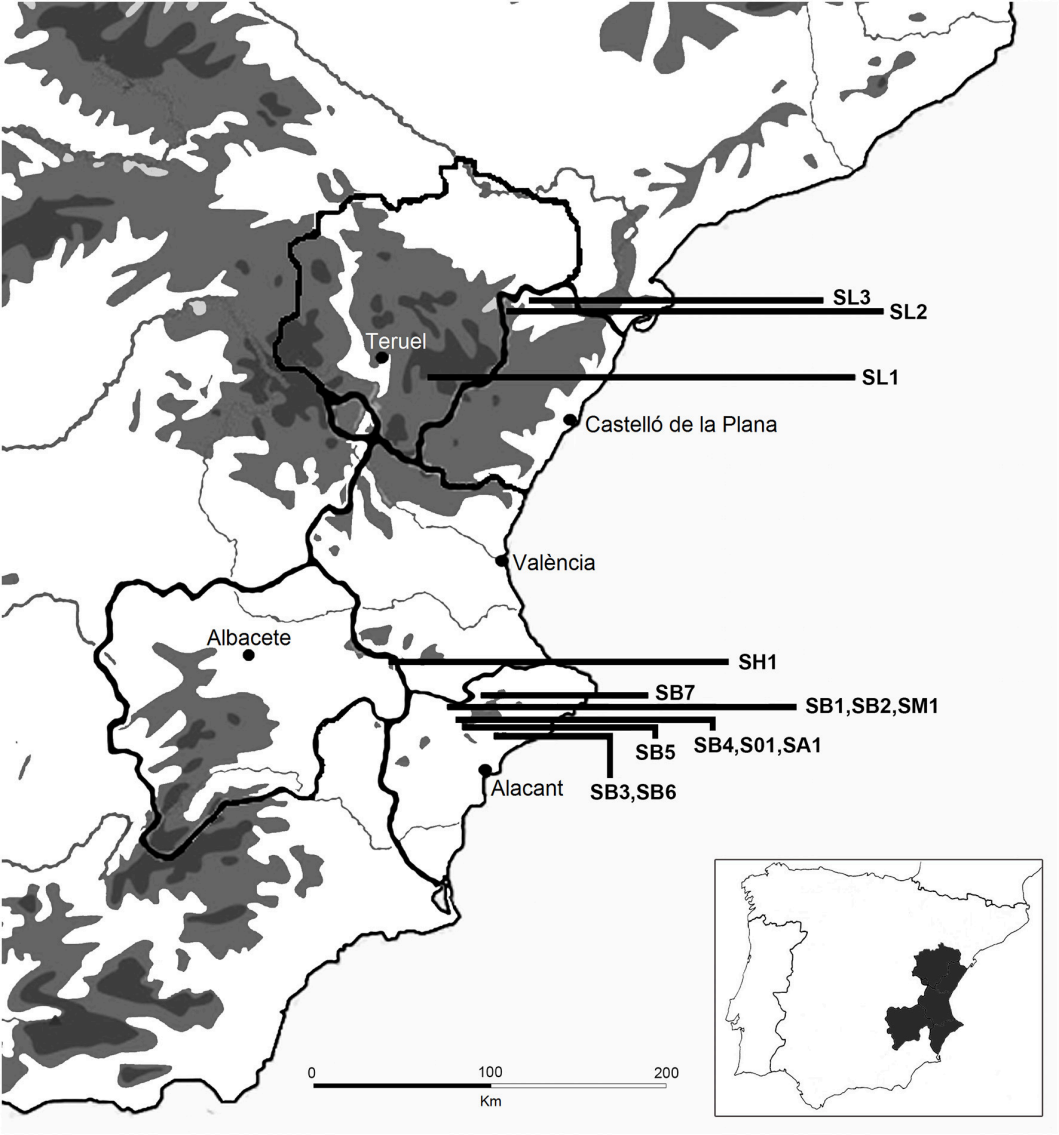
Map of the collection sites of the *Salvia* taxa studied (for identification codes, see Table 1).

This study has been carried along the mountainous areas of Valencia region (which includes the administrative provinces of Castellón, Valencia and Alicante) and bordering ones of Aragón, Castilla-La Mancha and Murcia regions. The region, located at the East and Southeast of Spain, comprises a thin coastal strip with wetlands and a fertile plain giving way in a few kilometers to a craggy inland territory in the West, with some high peaks (Figure 1).

### Ethnopharmacological research

The ethnopharmacological field study was carried out between 2009 and 2012, with semi-structured interviews with male and female informants randomly selected. The study was carried out in accordance with the recommendations of the Code of Ethics of the International Society of Ethnobiology (2006). Ethics approval was not required as per the author's institutions guidelines and national regulations. Verbal informed consent was obtained from each informant prior the interview. In this process, the objectives of the research, the topics and procedures for the interview and the general outcomes expected were presented to the informant and both confidentiality and complete anonymity were guaranteed. The consent of the informant was audio recorded. Only non-sensitive personal data were collected. Although elders were the most interesting informants, a few younger people were also interviewed in order to determine the intergenerational transmission of herbal knowledge (Martínez-Francés et al., 2012). Literature search for additional ethnopharmacological data has also been performed.

### Plant material

Aerial parts of six populations of *Salvia blancoana* subsp. *mariolensis*, two of *S. officinalis* subsp. *lavandulifolia*, and one of *S. x hegelmaieri, S. officinalis* subsp. officinalis, *S. x auriculata*, and *S. microphylla* var. *microphylla* were collected during flowering stage in June-July 2010 in different localities from Valencia region (Spain). In addition, two more samples, one of *S. blancoana* subsp. mariolensis and another of *S. officinalis* subsp. lavandulifolia were collected in winter time, with the aim of detecting seasonal changes in their essential oil composition (Table 1, Figure 1). Plant material was air-dried at room temperature in a dark, dry place and later stored until used.

**Table 1:** Collection data and essential oil yield for the aerial pats of the *Salvia* taxa studied.

**Code**	**Taxon**	**Localities**	**Origin**	**Collection date**	**Voucher specimen**	**Essential oil yield (% v/w)**
SB1	*S. blancoana* subsp. *mariolensis*	Banyeres de Mariola (Alacant, Spain)	Cultived from wild	06/06/2010	BCN 111823	4.4
SB2	*S. blancoana* subsp. *mariolensis*	Banyeres de Mariola (Alacant, Spain)	Cultived from wild	20/06/2010	BCN 111824	4.5
SB3	*S. blancoana* subsp. *mariolensis*	Font de l'Albre, Serra d'Aitana (Alacant, Spain)	Wild	29/06/2010	BCN 111825	2.7
SB4	*S. blancoana* subsp. *mariolensis*	Font de Vivens, Ibi (Alacant, Spain)	Wild	23/05/2010	BCN 111826	1.8
SB5	*S. blancoana* subsp. *mariolensis*	Torretes, Ibi (Alacant, Spain)	Wild	29/06/2010	BCN 111827	2.0
SB6	*S. blancoana* subsp. *mariolensis*	Port de Tudons, Alcoleja (Alacant, Spain)	Wild	29/06/2010	BCN 111828	3.0
SB7	*S. blancoana* subsp. *mariolensis*	Borona, Cocentaina (Alacant, Spain)	Wild	07/01/2011	BCN 111829	3.8
SH1	*S*. x *hegelmaieri*^a^	Sierra del Mugrón, Almansa (Albacete, Spain)	Wild	04/07/2010	BCN 111831	2.5
SL1	*S. officinalis* subsp. *lavandulifolia*^b^	Mora de Rubielos (Teruel, Spain)	Wild	20/06/2010	BCN 111832	1.2
SL2	*S. officinalis* subsp. *lavandulifolia*^b^	Cinctorres (Castelló, Spain)	Wild	01/07/2010	BCN 111833	1.2
SL3	*S. officinalis* subsp. *lavandulifolia*^b^	Herbés (Castelló, Spain)	Wild	06/12/2010	BCN 111830	1.3
SO1	*S. officinalis* subsp. *officinalis*	Torretes, Ibi (Alacant, Spain)	Landrace	20/06/2010	BCN 111834	2.4
SA1	*S*. x *auriculata*^c^	Torretes, Ibi (Alacant, Spain)	Landrace	29/06/2010	BCN 111835	1.9
SM1	*S. microphylla* var. *microphylla*	Banyeres de Mariola (Alacant, Spain)	Landrace	04/07/2010	BCN 111836	0.8

a *S. officinalis subsp. lavandulifolia x S. blancoana subsp. mariolensis*.

b *Syn. S. lavandulifolia subsp. lavandulifolia*.

c *S. officinalis subsp. officinalis x S. fruticosa*.

### Morphological identification

Identification of plant material was carried out using a stereomicroscope with the aid of literature data (Bolòs and Vigo, 1995; Laguna et al., 1998; López, 2001; Reales et al., 2004; Serra, 2007; Sáez, 2010; Mateo and Crespo, 2014). A voucher of each sample was deposited in the Herbarium BCF of the University of Barcelona (Barcelona, Spain) (Table 1).

### Chemicals and reagents

Anisaldehyde (Ref.: A88107), diphenylboric acid aminoethyl ester (Ref.: 42810), PEG 400 (Ref.: P3265), and rutin (Ref.: 78095) were obtained from Sigma Chemical Company (St. Louis, MO, USA). Eupatorin was from our own laboratory collection (previously isolated from *Salvia officinalis* subsp. *lavandulifolia* leaves). Acetic acid, formic acid, sulfuric acid, dichloromethane, ethanol, ethyl acetate, methanol, and toluene were of analytical grade and purchased from Panreac Química (Barcelona, Spain). HPTLC glass plates coated with Silicagel 60F_254_ (20 × 10 cm) were purchased from Merck (Darmstadt, Germany; Ref.: 1.05642.0001).

### Extracts

#### Essential oil isolation

Air-dried plant material of each sample (50 g) was submitted to hydrodistillation for 2.5 h using a Clevenger-type apparatus, as described in the European Pharmacopeia (EDQM, 2016). Essential oils were stored in amber colored glass vials at 4°C until analysis. Essential oil yields were determined using the same apparatus and 1,2,3,4-tetramethylbenzene as collector solvent, and were calculated as the mean of three replicates.

#### Preparation of extracts and reference solutions

Two types of extracts were obtained. Powdered air-dried plant material (0.5 g) was sonicated for 15 min with either 5 mL of ethanol/water (50/50, v/v) or 5 mL of dichloromethane. After centrifugation for 10 min, the extracts were filtered through a membrane filter of 45 μm and the supernatants, which were used as test solutions, were stored at 4°C till used.

Reference solutions of eupatorin and rutin used for HPTLC analysis of dichloromethane and hydroalcoholic extracts, respectively, were prepared at a concentration of 3 mg/10 mL in methanol and stored at 4°C till used.

### Analysis of the essential oil

#### GC-FID and GC-MS analysis

Essential oil analyses were carried out by GC-FID and GC-MS using two fused silica capillary columns (60 m × 0.25 mm i.d.; 0.25 μm film thickness) of different stationary phases: Supelcowax™10 and Equity™-1 (both from Supelco, Bellefonte, PA, USA).

GC-FID analyses were performed on a Hewlett-Packard (Palo Alto, CA, USA) model 6890 instrument, equipped with a HP GC ChemStation data processor software (version A.07.01), using the following analytical conditions: carrier gas, helium; flow rate, 1 mL/min; oven temperature programmed, 60°C (2 min), 60–180°C at 2°C/min, 180°C (7 min), 180–230°C at 4°C/min, 230°C (15 min), 230–260°C at 10°C/min, 260 °C (8 min); injector temperature, 250°C; detector temperature, 270°C; split ratio 1:100. A volume of 0.1 μL of the undiluted oils was injected.

Mass spectra were obtained with a computerized system constituted by a GC Hewlett-Packard 6890 gas chromatograph coupled to a mass selective detector Hewlett-Packard 5973N and equipped with GC-MS ChemStation software (version G1701DA), using the same analytical conditions as above. Transfer line temperature was 280°C. Mass spectra were taken over m/z 35–400, using an ionizing voltage of 70 eV.

#### Identification and quantification

Identification of essential oil constituents was achieved by means of their GC linear retention indices (RI) in the two stationary phases, determined in relation to a homologous series of *n*-alkanes (8–23 carbons) and a homologous series of fatty acid methyl esters (methyl esters of fatty acids with 4, 6, 8, 10, 12, 14, 16, and 18 carbon atoms, FAME indices), and comparison of fragmentation patterns in the mass spectra with those stored in our own library or in the GC-MS database and with literature data (Mc Lafferty, 1993; Adams, 2007). Quantification of each compound was performed on the basis of their GC-FID peak areas on the two columns, using the normalization procedure without corrections for response factor.

### HPTLC analysis

Hydroalcoholic and dichloromethane extracts were analyzed by HPTLC using a CAMAG (Muttenz, Switzerland) system constituted by a semiautomatic TLC Sampler Linomat 5 as application device, an Automatic Development Chamber ADC 2, a TLC Plate Heater III device, a Chromatogram Immersion Device III, Reprostar 3 (Digistore) as chromatogram documentation device, and the software WinCATS 1.4.6.

Test solutions were the hydroethanolic and dichlorometane extracts. Aliquots of 4 μL of test and reference solutions were applied on the plates as bands of 8 mm in length (distance from the left edge 15 mm and from the lower edge 8 mm). Plates were developed in ascending mode over a distance of 6 cm from the application position using a twin-through chamber fitted with filter paper previously saturated for 20 min with the mobile phase. Analyses were carried out at constant humidity (33%) at room temperature (23 ± 2°C). Two mobile phases were used depending on the test solutions analyzed: ethyl acetate, acetic acid, formic acid, water (100:10:10:25) for hydroethanolic extracts, and toluene, ethyl acetate, formic acid (70:30:1) for dichloromethane extracts.

Derivatization of the plates was carried out by dipping with either the Natural Products Reagent (NPR) and the PEG-400 solution (hydroethanolic extracts), or the anisaldehyde solution (dichloromethane extracts). In the case of hydroethanolic extracts, after elution, the plates were dried in cold air for 5 min, heated at 100°C for 3 min and dipped in the NPR solution (1 g of diphenylboric acid aminoethyl ester in 200 mL of ethyl acetate) while still hot. Once the plates were dried, they were subsequently dipped in the PEG-400 solution (10 g of PEG 400 in 200 mL of dichloromethane) and dried. Evaluation was performed under UV light at 366 nm. In the case of dichloromethane extracts, plates were immersed in anisaldehyde solution, prepared according to the European Pharmacopoeia (EDQM, 2016), and heated at 100°C for 5 min. They were examined immediately in daylight and eventually under UV light at 366 nm.

## Results and discussion

### Ethnopharmacological results

The ethnopharmacological study focused on traditional medicinal uses in Valencia region and closest mountainous areas by means of 87 interviews (2009–2012), 61 (33 men and 29 women) in the Valencia region, 19 (men) in Murcia and 6 (4 men and 2 women) in Aragón. The informants interviewed were aged between 24 and 83 years, being 61 the media in both sexes in Valencia region. In this area, nine Salvia species are used in folk medicine (Martínez-Francés et al., 2012). After a botanical identification of the different species used, some samples were collected for performing the chemical characterization (Table 1). The main focus of the present work is on *S. blancoana* subsp. *mariolensis* and *S. officinalis* subsp. *lavandulifolia*. For this reason, own and literature ethnopharmacological data of these two species is summarized in Table 2. Twenty nine medicinal uses were recorded for *S. officinalis* subsp. *lavandulifolia*, with a wider distribution area, from which 13 were also shared by *S. blancoana* subsp. *mariolensis*.

**Table 2:** Ethnopharmacological evidence for medicinal uses for *Salvia blancoana subsp. mariolensis* and *S. officinalis* subsp. *lavandulifolia* in Valencia region and surrounding areas.

**Diseases and related health problems^a^**	**Species^b^**	**Region^c^**	**Parts used^d^**	**Administration^e^**	**Preparation**	**Alleged activity (data source^f^)**
**I CERTAIN INFECTIOUS AND PARASITIC DISEASES**						
B35.3 Tinea pedis (Athlete foot)	SL	Ar	L	TOP	Herbal washes	Smelly feet (1)
**IV ENDOCRINE, NUTRITIONAL AND METABOLIC DISEASES**						
E70-E90 Metabolic disorders	SL	Ar, M	L	OR	Herbal tea	Depurative (2)
E70-E90 Metabolic disorders	SB	V	L	OR	Herbal tea	Detoxifying (1)
**VI DISEASES OF THE NERVOUS SYSTEM**						
G43.9 Migraine, unspecified	SL	Cs	AP, L	NAS	Vapor inhalation	Antimigraine (3)
**VII DISEASES OF THE EYE AND ADNEXA**						
H05.00 Unspecified acute inflammation of orbit	SL	Cs	AP, L	NAS	Vapor inhalation	Ophthalmic anti-inflammatory (3)
**IX DISEASES OF THE CIRCULATORY SYSTEM**						
I10-I15 Hypertensive diseases	SB	A, V	L	OR	Herbal tea	Hypotensive (1, 4, 5)
I10-I15 Hypertensive diseases	SL	Ab, Cs, V	AP, L	OR	Herbal tea	Hypotensive (1, 3, 6)
I99 Other and unspecified disorders of circulatory system	SL	Ar, M	L	OR	Herbal tea	To improve blood circulation (2)
**X DISEASES OF THE RESPIRATORY SYSTEM**						
J02.9 Acute pharyngitis, unspecified	SL	M	L	OR	Herbal tea	Pharyngitis (2)
J20 Acute bronchitis	SB	A,V	AP, L	OR	Herbal tea	Antitussive (1, 4)
J20 Acute bronchitis	SB	A,V	AP, L	OR	Herbal tea	Anti-cold (1, 4, 5)
J20 Acute bronchitis	SL	Cs, V	AP, L	OR	Herbal tea	Antitussive (1, 3)
J20 Acute bronchitis	SL	Ab, Cs, V	AP, L	OR	Herbal tea	Anti-cold (1, 3, 6, 7)
J45.9 Unspecified asthma	SL	M	L	OR	Herbal tea	Asthma (2)
**XI DISEASES OF THE DIGESTIVE SYSTEM**						
K03.6 Deposits on teeth	SL	Ab, M	L	OR	Masticatory	Teeth cleaning (1, 6)
K05.10 Gingivitis	SB	A	AP	OR	Herbal tea (mouthwash)	Against gum inflammation (5)
K30 Dyspepsia	SB	A, V	AP, L	OR	Herbal tea	Digestive (1, 4)
K30 Dyspepsia	SL	Ab, Cs, M, V	AP, L	OR	Herbal tea	Digestive (1, 2, 6)
K31.9 Disease of stomach and duodenum, unspecified	SL	M	L	OR	Masticatory	Stomach (2)
K52.3 Indeterminate colitis	SB	A, V	AP, L	OR	Hydroalcoholic extract	Tonic-digestive (1)
K52.3 Indeterminate colitis	SL	Cs, V	AP, L	OR	Hydroalcoholic extract	Tonic-digestive (1)
**XII DISEASES OF THE SKIN AND SUBCUTANEOUS TISSUE**						
L30.9 Dermatitis, unspecified	SL	Cs	AP, L	TOP	Cutaneous liquid wash	Anti-inflammatory (3)
L30.9 Dermatitis, unspecified	SL	Cs	AP, L	TOP	Hydroalcoholic extract	Anti-inflammatory (3)
**XIII DISEASES OF THE MUSCULOSKELETAL SYSTEM AND CONNECTIVE TISSUE**						
M15-M19 Osteoarthritis	SL	Cs	AP, L	NAS	Vapor inhalation	Anti-rheumatic (3)
**XIV DISEASES OF THE GENITOURINARY SYSTEM**						
N20. Calculus of kidney	SL	M	L	OR	Herbal tea	Kidney stones (2)
N94.6 Dysmenorrhoea, unspecified	SB	A, V	AP, L	OR	Herbal tea	Emmenagogue (1, 4)
N94.6 Dysmenorrhoea, unspecified	SL	Ab, Ar, Cs, M, V	AP, L		Herbal tea	Emmenagogue (1, 3)
**XVIII SYMPTOMS, SIGNS AND ABNORMAL CLINICAL AND LABORATORY FINDINGS, NOT ELSWHERE CLASSIFIED**						
R07.0 Pain in throat	SL	M	L	OR	Masticatory	Throat pain (2)
R07.0 Pain in throat	SL	Ab	AP	OR	Herbal tea (gargle)	Throat pain (6)
R14 Flatulence and related conditions	SB	A, V	AP, L	OR	Herbal tea	Digestive (1, 4)
R14 Flatulence and related conditions	SL	Ab, Cs, V	AP, L	OR	Herbal tea	Digestive (1)
R45.0 Nervousness	SB	A, V	AP, L	NAS	Vapor inhalation	Nervous sedative (1)
R45.0 Nervousness	SB	A, V	AP, L	OR	Herbal tea	Nervous sedative (1)
R45.0 Nervousness	SL	Cs, V, M	AP, L	OR	Herbal tea	Nervous sedative (1, 2, 3)
R49.1 Aphonia	SL	Ab	AP	OR	Herbal tea (gargle)	Aphonia (6)
R50.9 Fever, unspecified	SB	A,V	AP, L	OR	Herbal tea	Antipyretic (1)
R50.9 Fever, unspecified	SL	Cs, V, M	AP, L	OR	Herbal tea	Antipyretic (1)
R51 Headache	SL	M	L	TOP	Fresh leaves (rubbed on the forehead)	Headache (2)
R53.83 Other fatigue	SL	Ab	AP	TOP	Alcoholic maceration (rubbed on the back and chest)	Against fatigue (6)
R61 Generalized hyperhidrosis	SB	A,V	L	TOP	Cutaneous liquid wash	Anti-hyperhidrosis (1)
R61.0 Localized hyperhidrosis	SL	Ar	L	TOP	Herbal washes	Smelly feet (1)
R63.0 Loss of appetite. Anorexia	SL	M	L	OR	Herbal tea	Appetizer (2)
**XIX INJURY, POISONING AND CERTAIN OTHER CONSEQUENCES OF EXTERNAL CAUSES**						
T63.2 Toxic effect of contact with scorpion	SL	Cs	AP	TOP	Poultice	Antidote for scorpion bites (3)

a *Pathologies were standardized following the International Statistical Classification of Diseases and Related Health Problems 10th Revision (ICD-10) Version for 2010 (WHO, 2017)*.

b *SB, Salvia blancoana subsp. mariolensis; SL, S. officinalis subsp. lavandulifolia*.

c *A, Alicante province; Ab, Albacete province; Ar, Aragon region; Cs, Castellón province; M, Murcia; V, Valencia province*.

d *AP, aerial parts; L, leaves*.

e *NAS, nasal and upper respiratory tract; OR, oral; TOP, topic*.

f *1, Own data, including Martínez-Francés et al. (2012); 2, Obón and Rivera (1991); 3, Mulet (1991); 4, Pellicer (2001); 5, Pérez (2011); 6, Verde et al. (2008); 7, Fresquet et al. (1994)*.

In Valencia region, *S. blancoana* subsp. *mariolensis* is often used to treat diseases of the circulatory, respiratory, digestive and genitourinary systems. Its aerial parts are commonly used as a tea, alone or mixed with other species. Informants believe that these preparations are hypotensive, antitussive or anti-cold, tonic-digestive, emmenagogue, febrifuge, sedative, and detoxifying. The local vernacular names are *sàlvia, sàlvia de la Mariola, sàlvia de serra, sàlvia blanca*, and *savia* (Pérez, 2011; Martínez-Francés et al., 2012) and it is considered the best sage for medicinal purposes.

*Salvia officinalis* subsp. *lavandulifolia* has similar uses in Valencia region, where it is known as *sàlvia, sàlvia de serra, sàvia, sava, sèrvia*, and *sèlvia*. Infrequent uses, are related to eye, skin, or the musculoskeletal or nervous system. In Castellón province, it is used in inhalation fumes for migraines, as ophthalmic antiinflamatory and for rheumatism. In the Aragonese borderlands, this sage is less frequent and less cited, although it is highly prized; it is known as *sàlvia, sàlvia d'Aragó, estepera*, and as *salvia de Penyagolosa* (mountain of Valencia region where it is collected). The main traditional uses in Aragon are as digestive and depurative, and it is considered very effective against menstrual problems and to eliminate the bad odor of the feet. In Murcia region, *S. officinalis* subsp. *lavandulifolia* is locally known as *manisierva, manisielva, marisielva, mariselva, mariserva, marisierva, salvia*, and *savia*. It is used for the same purposes cited above, but also as aperitif (Obón and Rivera, 1991) and as masticatory to clean the teeth. The last use is also found in Albacete (Castilla—La Mancha), where, macerated in alcohol, is additionally employed for rubbing back and chest to relieve fatigue. The decoction has been used as gargle for snoring and sore throats (Verde et al., 2008). In Albacete, bordering Valencia region, this species and the hybrid *S*. x *hegelamieri*, are indistinctly employed. For *S. officinalis* subsp. *lavandulifolia*, some popular names, *marisielva, marisilva, salvia, savia, selva, sielva*, and *sierva*, resemble to those collected in Murcia and Valencia regions.

The alcoholic preparation known as “*salvieta”* or “*licor de salvia”* (sage liquor), which is used as digestive, is especially made in *S. blancoana* subsp. *mariolensis* distribution area (Martínez-Francés et al., 2012). “*Salvieta*” is a homemade anise-flavored alcoholic maceration of the aerial parts of *S. blancoana* subsp. *mariolensis*, alone or, rarely, mixed with other sages, such as, *S. officinalis* subsp. *officinalis, S. officinalis* subsp. *lavandulifolia*, and *S. microphylla* var. *microphylla*. Nowadays this local product has not a great demand outside the zone of preparation, but some small industries of this area are already marketing the raw material for teas or as a kit for a homemade liquor elaboration. However, there is a potential market for this sage liquor, because its consumption in local festivities and annual fairs are consolidating its use among the youngest. To prevent adulterations, a correct identification of the raw material is necessary.

### Morphological characterization

It is a common criterion to consider *S. officinalis* subsp. *officinalis*, traditionally cultivated in Spain for centuries, as an introduced species from the Eastern Mediterranean along with other species, such as, *S. fruticosa* and *S. x auriculata* Mill., an hybrid of both (Reales et al., 2004). Concerning *S. officinalis* subsp. *lavandulifolia*, its independence (as *S. lavandulifolia*) was recognized by several authors. Nevertheless, Bolòs and Vigo (1995) and, more recently, Reales et al. (2004) subordinated it to *S. officinalis*, mainly due to the high coincidence of the indumentum of the calyx. This criterion is nowadays internationally accepted (TPL, 2017) and has been followed in the present work. *S. microphylla* var. *microphylla*, a foreign species coming from Mexico (Valdés et al., 1995), cultivated in this area for more than 150 years, is also used in Valencian folk medicine together with *S. officinalis* subsp. *officinalis, S. officinalis* subsp. *lavandulifolia, S. blancoana* subsp. *mariolensis*, and *S. x auriculata*. All of them have been collected for morphological and chemical characterization.

As it has been exposed above, discrepancies between Spanish botanists on the classification of *Salvia* taxa greatly complicate the identification of the raw material for the liquor and herbal markets, in which an unambiguous identification should be a priority before commercialization. In order to avoid misunderstanding, the taxonomic treatment proposed by Reales et al. (2004) and Sáez (2010) is followed here. *Salvia officinalis* related sages present in the Iberian Peninsula are organized in seven taxa: *Salvia fruticosa* Mill., *S. officinalis* L. subsp. *officinalis, S. officinalis* L. subsp. *lavandulifolia* (Vahl) Gram, *S. officinalis* L. subsp. *oxyodon* (Webb & Heldr.) Reales, D.Rivera & Obón, *S. blancoana* Webb and Heldr. subsp. *blancoana, S. blancoana* Webb & Heldr. subsp. *vellerea* (Cuatrec.) W. Lippert, *S. blancoana* Webb and Heldr. subsp. *mariolensis* Figuerola, and two of their hybrids: *S*. x *auriculata* Mill. (*S. officinalis* subsp. *officinalis* x *S. fruticosa*) and *S*. x *hegelmaieri* Porta Rigo (*S. officinalis* subsp. *lavandulifolia* x *S. blancoana* subsp. *mariolensis*).

Macro and micromorphological characters are useful for the identification of the different Iberian *Salvia* subgenus *Salvia* taxa related to *S. officinalis*. As it is shown in Table 3, leaves of *S. blancoana* subsp. *mariolensis* are oblanceolate, while those of *S. officinalis* subsp. *lavandulifolia* are linear-lanceolate and those of *S. officinalis* subsp. *officinalis* and *S. fruticosa* are ovate. *Salvia blancoana* subsp. *mariolensis* present persistent upper floral bracts in contrast with the rest. The presence of cup-shaped glandular trichomes and long patent eglandular trichomes can separate it from *S. officinalis* subsp. *lavandulifolia. Salvia blancoana* subsp. *mariolensis* and *S. blancoana* subsp. *vellerea*, with different distribution areas, exhibit different type of leaves, the second lacking sessile glands. *Salvia microphylla* var. *microphylla* (subgenus *Calosphace*), is not included in Table 3 due to its distinct morphology, especially its delta-shaped leaves and red flowers, which clearly differentiates it from the other sages. The raw herbal material from all these taxa is frequently dried and cut before marketed, making even more difficult its identification. Therefore, other techniques, such as, chemical profiling, are useful.

**Table 3:** Main macro and micromorphological characters for identification of the aerial parts of some taxa of *Salvia* traditionally used in the Iberian Peninsula^a^.

**Taxon^b^**	**Calix shape**		**Calix trichome**				**Upper floral bracts**	**Leaves**	
	**Simetry**	**Reticulate between veins**	**Eglandular**	**Glandular**				**Type**	**Shape**
				**Sessile^c^**	**Stalked**	**Cup-shaped^d^**			
*S. fruticosa* Mill.	Actinomorphic	No	Abundant (long patent)	Absent	Abundant	Abundant	Always deciduous	Simple, eared or trilobated	Ovate to obovate
*S. officinalis* L. subsp. *officinalis*	Bilabiate	Strongly	Scarce	Abundant	Absent	Absent	Always deciduous	Simple	Ovate to obovate
S. *officinalis* L. subsp. *lavandulifolia* (Vahl) Gams	Actinomorphic or slightly bilabiate	No	Abundant (short antrorse)	Abundant	Absent	Absent	Always deciduous	Simple	Linear-lanceolate to ovate
*S. officinalis* L. subsp. *oxyodon* (Webb & Heldr.) Reales, D.Rivera & Obón	Actinomorphic or slightly bilabiate	No	Rare	Abundant	Absent	Absent	Always deciduous	Simple	Lanceolate
*S. blancoana* Webb & Heldr. subsp. *blancoana*	Actinomorphic or slightly bilabiate	No	Scarce	Abundant	Absent	Abundant	Persistent or deciduous	Simple	Oblanceolate to ovate-lanceolate
*S. blancoana* Webb & Heldr. subsp. *vellerea* (Cuatrec.) W.Lippert	Actinomorphic or slightly bilabiate	No	Abundant (long patent)	Scarce	Absent	Abundant	Persistent or deciduous	Simple (rarely trilobated)	Oblanceolate to ovate-lanceolate
*S. blancoana* Webb & Heldr. subsp. *mariolensis* Figuerola	Actinomorphic or slightly bilabiate	No	Abundant (long patent)	Abundant	Absent	Abundant	Persistent or deciduous	Simple	Oblanceolate

a *Prepared from Reales et al. (2004), Sáez (2010) and our own data*.

b *Hybrids as S. x auriculata (S. officinalis subsp. officinalis x S. fruticosa) and S. x hegelmaieri (S.officinalis subsp. lavandulifolia x S. blancoana subsp. mariolensis) have intermediate characters of their parental*.

c *Equivalent to glandular trichomes of lamiaceous type, according to European Pharmacopeia (EDQM, 2016, 9th Edition)*.

d *Long glandular trichomes with their secretory material lost. The view corresponds to their stalk: ‘Type 2’ in Reales et al. (2004)*.

### Essential oil content and composition

In order to contribute to the chemical characterization of *S. blancoana* subsp. *mariolensis*, the essential oil contents of the air-dried aerial parts of seven samples of this taxon have been determined and compared with those obtained for samples of other sages used in the region (Table 1). The essential oil content (% v/w) of *S. blancoana* subsp. *mariolensis* was in general higher than 2% (1.8–4.5%), even for a sample collected in winter (SB7), which yielded 3.8% of oil. Only one sample (SB4) gave a lower yield (1.8%). The essential oil contents of the three samples of *S. officinalis* subsp. *lavandulifolia* were always below 2% (1.2–1.3%) and lower than those shown by *S. blancoana* subsp. *mariolensis*. Finally, their hybrid, *S. x hegelmaieri*, gave a content of 2.5%, which is in between of those shown by the parent taxa.

It is the first time that the detailed composition of the essential oil of *S. blancoana* subsp. *mariolensis* is described. Only some preliminary data were previously published by us (Martínez-Francés et al., 2012). In the present work, the essential oils distilled from the air-dried aerial parts of seven samples of this taxon (SB1–SB7), collected in different locations and including wild and cultivated plant material, were submitted to exhaustive analysis by GC-FID and GC-MS. The results, which are shown in Table 4 (main constituents) and Supplementary Table 1 (complete list of constituents), were compared with those given by the oils of three samples of *S. officinalis* subsp. *lavandulifolia* (SL1–SL3). In addition, the composition of the essential oil of the aerial parts of *S*. x *hegelmaieri*, the hybrid of the two former taxa growing in the same geographical area, is reported for the first time (Table 4, Supplementary Table 1).

**Table 4:** Composition of the essential oils of *Salvia blancoana* subsp. *mariolensis, S*. x *hegelmaieri* and *S. officinalis* subsp. *lavandulifolia* from the Valencia region (Spain) and close areas^a^.

**Retention indices^b^**				**Constituents^c^**	**Percentage in the essential oils^d^**											**Identification methods^b^^,^^e^**
**A**	**B**	**C**	**D**		**SB1**	**SB2**	**SB3**	**SB4**	**SB5**	**SB6**	**SB7**	**SH1**	**SL1**	**SL2**	**SL3**	
1,030	933	115	211	α-Pinene	3.6	3.0	4.2	6.5	4.9	7.4	4.3	6.0	3.7	9.5	3.1	A,B,C,D,E
1,077	946	135	218	Camphene	4.2	6.0	4.7	6.0	5.6	8.5	4.6	5.8	3.8	5.6	7.6	A,B,C,D,E
1,118	973	156	229	β-Pinene	5.0	7.6	5.7	6.3	6.0	5.1	5.8	5.8	3.1	6.3	7.7	A,B,C,D,E
1,163	984	184	233	Myrcene	5.6	10.0	3.0	5.0	3.7	5.4	4.0	4.3	3.7	5.0	2.5	A,B,C,D,E
1,205	1,026	207	254	Limonene	1.8	1.2	2.1	2.7	1.7	5.0	4.5	3.3	3.3	6.7	8.2	A,B,C,D,E
1,220	1,026	213	254	1,8-Cineole	40.4	40.1	32.0	25.4	35.3	13.7	45.7	40.0	27.1	19.8	25.6	A,B,C,D,E
1,236	1,027	221	258	*cis*-β-Ocimene	1.1	0.4	3.3	2.4	2.6	3.5	1.6	0.1	0.1	0.2	1.5	A,B,C,D,E
1,280	1,015	241	248	*p*-Cymene	0.5	0.1	0.8	0.9	0.3	1.0	0.8	0.4	2.0	2.5	3.3	A,B,C,D,E
1,536	1,126	368	304	Camphor	21.1	19.6	24.4	20.3	18.8	28.6	12.1	15.9	23.9	16.2	16.8	A,B,C,D,E
1,592	1,270	395	381	Bornyl acetate	1.5	0.3	1.6	1.9	1.9	3.1	0.1	1.4	1.5	2.0	0.1	A,B,C,D,E
1,610	1,417	403	458	β-Caryophyllene	0.2	0.7	1.5	3.5	1.2	0.4	0.4	0.4	3.6	2.4	0.6	A,B,C,D,E
1,681	1,176	439	329	Estragole	−	*t*	−	−	−	−	−	0.8	0.8	2.2	−	A,B,C,D,E
1,708	1,332	451	417	α-Terpenyl acetate	0.6	−	0.2	0.6	2.3	**t**	−	0.1	*t*	0.1	*t*	A,B,C,D,E
1,713	1,153	454	318	Borneol	3.0	0.8	3.7	5.2	5.1	7.4	2.7	3.4	4.6	5.1	4.2	A,B,C,D,E
2,097	1,582	641	537	Viridiflorol	−	*t*	*t*	*t*	0.4	*t*	*t*	*t*	1.8	3.7	0.1	A,B,C,D,E
**COMPOSITION BY GROUPS OF CONSTITUENTS^f^**																
				Monoterpene hydrocarbons	24.7	31.3	27.3	32.3	27.7	39.3	28.3	28.8	21.3	38.2	41.1	
				Oxygenated monoterpenes	71.2	63.8	66.3	57.5	66.2	56.8	66.7	65.6	62.6	46.5	54.4	
				Sesquiterpene hydrocarbons	0.8	1.8	2.6	5.7	2.1	1.9	1.3	1.1	8.1	5.7	1.4	
				Oxygenated sesquiterpenes	1.8	1.1	2.0	2.8	2.3	1.6	2.6	2.0	5.8	5.9	2.5	
				Phenylpropanoids	−	*t*	*t*	−	−	*t*	−	1.4	0.8	2.3	*t*	
				Others	0.1	0.2	0.1	0.3	*t*	*t*	0.1	0.2	0.2	0.2	**t**	
				Total identified	98.5	98.2	98.3	98.5	98.3	99.6	98.9	99.1	98.9	98.8	99.3	

a *Only constituents with percentages higher than 2% in at least one of the essential oils are reported in this table. For complete list of constituents, see Supplementary Table 1*.

b *Retention indices: A, n-Alkane indices in Supelcowax™-10; B, n-Alkane indices in Equity-1™; C, FAME indices in Supelcowax™-10; D, FAME indices in Equity-1™*.

c *Compounds listed by elution order in the polar column (Supelcowax™-10) except the last fourteen constituents*.

d *For the meaning of the identification codes, see Table 1. t: traces (≤0.05%)*.

e *Identification method E: GC-MS*.

f *Calculated taking in account the complete list of constituents*.

More than 98% of each essential oil was identified, which, considering all the samples analyzed, involved a total of 174 constituents. In all cases, the main group of constituents were oxygenated monoterpenes (46.5–71.2%), followed by monoterpene hydrocarbons (21.3–41.1%). The essential oil of *S. blancoana* subsp. *mariolensis* is characterized by 1,8-cineole (13.7–45.7%) and camphor (12.1–28.6%) as major constituents, followed by myrcene (3.0–5.6%), α-pinene (3.0–7.4%), camphene (4.2–8.5%), β-pinene (5.0–7.6%), limonene (1.7–5.0%), and borneol (0.8–7.4%). Among sesquiterpenes (2.6–8.5%), β-caryophyllene (0.2–3.5%), β-caryophyllene oxide (0.3–1.2%), and spathulenol (0.2–1.3%) reached the higher percentages. The essential oil of *S*. x *hegelmaieri* showed a similar pattern, with 1,8-cineole (40.0%), camphor (15.9%), α-pinene (6.0%), camphene (5.8%), β-pinene (5.8%), myrcene (4.3%), borneol (3.4%), and limonene (3.3%) as the main constituents.

As it can be seen in Table 4, the essential oils of the three taxa analyzed showed a similar profile, mainly concerning their major constituents. They are, in general, in accordance with those previously reported for the essential oils from *S. officinalis* subsp. *lavandulifolia* (Lawrence et al., 1970; Marcos Sanz et al., 1988; Martínez-Francés et al., 2012; Usano-Alemany, 2012), with 1,8-cineole and camphor as major constituents. However, some minor distinguishing features could be recognized. In fact, *p*-cymene was found in the three samples of *S. officinalis* subsp. *lavandulifolia* in a range from 2.0 to 3.3%, while it did not reach 1% either in *S. blancoana* subsp. *mariolensis* or in *S*. x *hegelmaieri*. Furthermore, the phenylpropanoid estragole was detected in traces in only one sample from *S. blancoana* subsp. *mariolensis*, whereas it was found in *S*. x *hegelmaieri* (0.8%) and two samples of *S. officinalis* subsp. *lavandulifolia* (0.8 and 2.2%).

In order to investigate seasonal variations in the essential oil composition of *S. blancoana* subsp. *mariolensis* and *S. officinalis* subsp. *lavandulifolia*, two samples were collected at winter time (SB7 and SL3, respectively). In general, the chromatographic profile of the latter was inside the range found for spring-early summer samples of *S. officinalis* subsp. *lavandulifolia*. Nevertheless, in the case of *S. blancoana* subsp. *mariolensis*, the essential oil of the winter sample (SB7) showed a significantly higher percentage of 1,8-cineole and a lower percentage of camphor, the two major constituents, when compared to the essential oils from samples collected in late spring-early summer (SB1–SB6).

In addition to the similarity of the composition of the essential oils of the three taxa investigated, it is noteworthy to remark that they show chromatographic profiles that are mostly in accordance with those prescribed in the current standards for “Spanish sage oil” in the European Pharmacopoeia (EDQM, 2016) (monograph 1849), for “oil of sage, Spanish” by the International Organization for Standardization (standard ISO 3526) (ISO, 2005) and for “aceite esencial de salvia de España” by the Spanish Association for Standardization (AENOR, standard UNE 84310) (AENOR, 2001; Table 5). Minor differences can be observed, that could be due to the fact that the essential oils studied here were hydrodistilled in the laboratory from small amounts of dried plant material, whereas standards are established for essential oils obtained by steam distillation at industrial scale. The species accepted for the preparation of the essential oil in these standards is *S. lavandulifolia* (referred here as *S. officinalis* subsp. *lavandulifolia*). Nevertheless, according to our results, also *S. blancoana* subsp. *mariolensis* should be considered as a possible source of this oil. In fact, this endemic Levantine sage appears in a small mountainous area between northern Alicante and southern Valencia provinces, immersed within the wide distribution area of *S. officinalis* subsp. *lavandulifolia*. Therefore, when wildgrowing, these two sages could be collected without any distinction.

**Table 5:** Comparison of the composition found for the essential oils from *S. blancoana* subsp. *mariolensis, S*. x *hegelmaieri* and *S. officinalis* subsp. *lavandulifolia* to the current requirements for Spanish sage oil in the standards from the European Pharmacopoeia (EDQM, 2016) (monograph 1849), ISO (2005) (standard ISO 3526) and AENOR (2001) (standard UNE 84310).

**Component^a^^,^^b^**	***S. blancoana* subsp. *mariolensis***	***S*. × *hegelmaieri***	***S. officinalis* subsp. *lavandulifolia***	**UNE-84310 ISO 3526**	***Ph.Eur*. 9th edition**
α-Pinene	3.0−7.4	6.0	3.7−9.5	4−11	4.0−11.0
Sabinene	0.6−1.8	1.5	0.1–0.6	0.1−3.5	0.1−3.5
Limonene	1.2−5.0	3.3	3.3−6.7	2−6	2.0−6.5
1,8-Cineole	13.6−40.4	40.0	19.8−27.1	10−30	10.0−30.5
Linalool	0.2−0.9	0.3	0.2	0.3−4	0.3−4.0
Camphor	18.8−28.6	15.9	16.2−23.9	11−36	11.0−36.0
Borneol	0.9−7.4	3.5	4.6−5.1	1−7	1.0−7.0
Terpinen-4-ol	0.0−0.6	0.7	*t*−0.4	<2	<2.0
Linalyl acetate	0.0−0.3	**t**	0.0−t	0.1−5	<5.0
α-Terpenyl acetate	0.0−2.3	0.1	*t*−0.1	0.5−9	0.5−9.0
Sabinyl acetate	0.0−1.0	0.0	0.0−0.1	0.5−9	0.5−9.0
Thujone	0.0−*t*	0.0	0.0−*t*	−	<0.5

a *All values are in percentage, showing the range found or accepted for each constituent. In the case of S. x hegelmaieri, only one sample was analyzed*.

b *t = ≤ 0.05%*.

The essential oil of *S. officinalis* subsp. *lavandulifolia*, after a single oral dose of 50 μL, has shown to improve cognitive function in healthy humans compared to placebo, specially the performance of secondary memory and attention tasks (Kennedy et al., 2011). This effect has been mainly related to the inhibition of acetylcholinesterase by the oil, 1,8-cineol followed by α-pinene being the most active constituents (Perry et al., 2003; Kennedy et al., 2011). Taking in account the similarity in composition with the essential oil of *S. blancoana* subsp. *mariolensis*, which is particularly rich in 1,8-cineol, the effect on cognitive function could also be expected from the oil of this species.

Finally, it is important to remark that α-thujone and β-thujone are absent or only found in traces in the essential oils of *S. blancoana* subsp. *mariolensis, S. officinalis* subsp. *lavandulifolia*, and *S*. x *hegelmaieri*. Due to its activity on the central nervous system, thujone is considered toxic, especially the α isomer which is the most potent, and the control of its content in foods and medicines is necessary (Emmert et al., 2004). The low content/absence of thujone in the essential oils analyzed here is important in order to avoid any safety concern for this reason in relation to the herbal products and, specially, the liquors prepared thereof, since they will follow the recommendations of the European Medicines Agency for herbal medicinal products (EMA, 2011), and the regulations for alcoholic and non-alcoholic beverages established in the European Directive 1334/2008/EC (European Parliament and Council, 2008).

### HPTLC characterization

With the aim of setting a simple test allowing an easy distinction of plant material coming from different species, hydroalcoholic and dichloromethane extracts of the dried aerial parts of the taxa were analyzed by HPTLC according to the methods described in the experimental section. Two HPTLC systems with optimized mobile phases were developed for the analysis of each type of extract.

**Figure 2:**
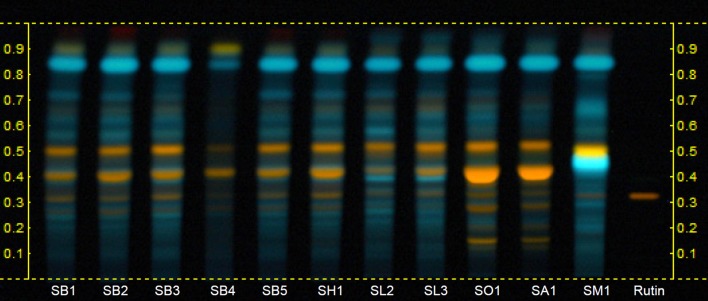
HPTLC fingerprints of hydroalcoholic extracts of the aerial parts of *Salvia* species used in Valencian folk medicine. Mobile phase*:* Ethyl acetate:formic acid:acetic acid:water (100:10:10:25). Derivatization: NPR + PEG 400, UV 366 nm. Track identification: SB1 to SB5, *S. blancoana* subsp. *mariolensis*; SH1, *S*. x *hegelmaieri*; SL2 and SL3, *S. officinalis* subsp. *lavandulifolia*; SO1, *S. officinalis* subsp. *officinalis*; SA1, *S*. x *auriculata*; SM1, *S. microphylla* var. *microphylla*.

Figure 2 shows the HPTLC chromatograms of the hydroalcoholic extracts examined under UV light at 366 nm after derivatization with NPR and PEG-400 solution. With this system, the chromatographic profiles of *S. blancoana* subsp. *mariolensis, S. officinalis* subsp. *lavandulifolia*, and *S*. x *hegelmaieri* were very similar to each other with only slight differences concerning the intensities of certain zones. The only remarkable difference is a fluorescent blue zone at Rf 0.4 present in the case of *S. officinalis* subsp. *lavandulifolia*, which is not observed in *S. blancoana* subsp. *mariolensis* nor in *S*. x *hegelmaieri*. High similarity is also observed between *S. officinalis* subsp. *officinalis* and *S. x auriculata*, which, nevertheless, could be distinguished from the former three thanks to the presence of one additional orange fluorescent zone in the lower third of the chromatogram and the much higher intensity of the lower of the two orange fluorescent zones present in the middle third. Finally, the hydroalcoholic extract of *S. microphylla* var. *microphylla* showed a clearly different chromatographic profile from all other taxa analyzed, mainly due to the presence, in the middle third, of one intense blue fluorescent zone (Rf 0.45) and a yellow fluorescent zone (Rf 0.49), which are absent in the other taxa. Thus, the fingerprints obtained with the hydroalcoholic extracts are not useful enough for the distinction of *S. blancoana* subsp. *mariolensis* from *S. officinalis* subsp. *lavandulifolia*. Although these two taxa have partially similar uses, they do not have overlapping distribution and the former constitutes a local resource for inland highland areas of Alicante province. For a potential commercialization and valorization of the traditional beverage “salvieta,” used only in this area, it is worth to set up a suitable HPTLC system for differentiating these two species.

**Figure 3:**
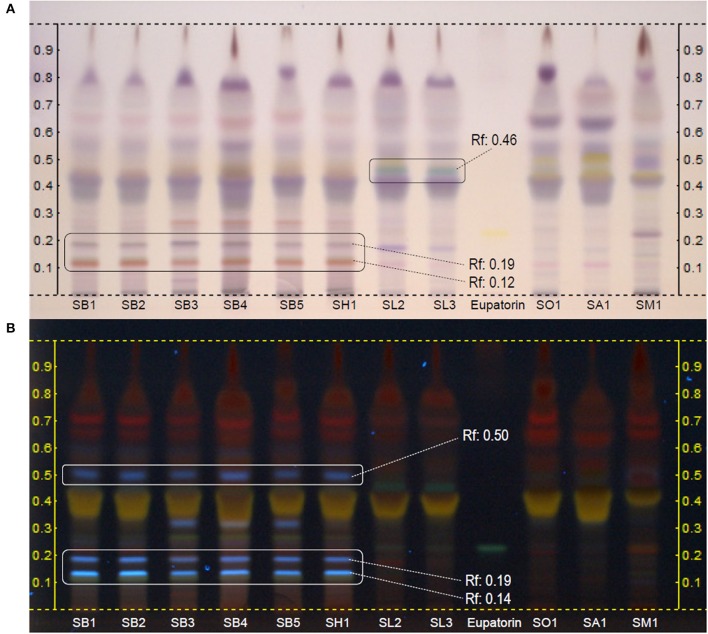
HPTLC fingerprints of dichloromethane extracts of the aerial parts of *Salvia* species used in Valencian folk medicine. Mobile phase*:* Toluene:ethyl acetate:formic acid (70:30:1). Derivatization*:* anisaldehyde reagent. Visualization: white light **(A)** and UV 366 nm **(B)**. Track identification: SB1 to SB5, *S. blancoana* subsp. *mariolensis*; SH1, *S*. x *hegelmaieri*; SL2 and SL3, *S. officinalis* subsp. *lavandulifolia*; SO1, *S. officinalis* subsp. *officinalis*; SA1, *S*. x *auriculata*; SM1, *S. microphylla* var. *microphylla*

The HPTLC analysis of dichloromethane extracts derivatized with anisaldehyde reagent and examined under daylight or UV 366 nm, provided characteristic fingerprints for most of the taxa analyzed, as it is shown in Figure 3. All samples of *S. blancoana* subsp. *mariolensis* (SB1-SB5) showed a homogeneous fingerprint, which was similar to the one obtained with *S. x hegelmaieri*, a hybrid of the former, botanically very close. These two taxa were clearly differentiated from the others by two zones observed at daylight, appearing in the lower part of the chromatogram (Figure 3A), at Rf 0.12 (red-brown) and Rf 0.19 (gray-blue). Furthermore, examination of the plate under UV light at 366 nm (Figure 3B) showed three blue fluorescent bands at Rf 0.12, Rf 0.14 and Rf 0.50 which were absent in the other taxa. Therefore, all these zones might be considered as specific markers for *S. blancoana* subsp. *mariolensis* and *S*. x *hegelmaieri*. In the case of *S. officinalis* subsp. *lavandulifolia*, a blue-green zone in the middle third (Rf 0.45) of the chromatogram observed under white light (Figure 3A) appears to be characteristic of this taxon. This zone is also seen under UV light at 366 nm as a greenish gray zone (Figure 3B). The fingerprints of the cultivated species *S. officinalis* subsp. *officinalis, S*. x *auriculata*, and *S. microphylla* var. *microphylla* showed visible differences after derivatization of the plates with anisaldehyde and observation under white light (Figure 3A).

## Conclusions

The traditional uses of *S. blancoana* subsp. *mariolensis* are close to those of *S. officinalis* subsp. *lavandulifolia*, but in a fewer number. Some morphological characters of the first species can discriminate it from the second one, as well as from other *Salvia* species. Nevertheless, identification using macro and micromorphological characters can be difficult when the raw herbal material is dried and cut, and is not possible in the case of extracts. In this context, the proposed HPTLC fingerprinting allows to discriminate the species and is suitable for quality control of the herbal material of *S. blancoana* subsp. *mariolensis* used in the preparation of herbal products and liquors.

The composition of the essential oils of *S. officinalis* subsp. *lavandulifolia, S. blancoana* subsp. *mariolensis* and their hybrid *S* x *hegelmaieri* is very similar, and mostly in accordance with the chromatographic profile prescribed in the current standards of the European Pharmacopeia, ISO and AENOR for the “Spanish sage oil.” Therefore, *S. blancoana* subsp. *mariolensis* and its hybrid should be considered as suitable sources of the oil together with the accepted *S. officinalis* subsp. *lavandulifolia*. There is no safety concern related to the content of thujone for the consumption of the traditional liquors homemade from these species, since this compound is only found in trace amounts in their essential oils.

## Author contributions

VM, SR, and DR were responsible for field work, interviews, collection and identification of samples and ethnopharmacological analysis. EH, VM, RV, ER, and SC designed and performed the chemical analysis. RV, VM, DR, and SC responsible for writing the manuscript. All authors proof read manuscript and made contributions to the final version.

### Conflict of interest statement

The authors declare that the research was conducted in the absence of any commercial or financial relationships that could be construed as a potential conflict of interest. The reviewer RM and handling Editor declared their shared affiliation, and the handling Editor states that the process nevertheless met the standards of a fair and objective review.
