# Supplementary material for: Ethnopharmacological and Chemical Characterization of Salvia Species Used in Valencian Traditional Herbal Preparations
Source: Front Pharmacol. 2017 Jul 25;8:467. doi: 10.3389/fphar.2017.00467 (PMC5524814; doi:10.3389/fphar.2017.00467)
Supplement: Supplementary file 1 [file Table1.pdf]

Martínez-Francés *et al.* (2017) *Front. Pharmacol.* 8:467.
doi: 10.3389/fphar.2017.00467

Supplementary Material

Ethnopharmacological and chemical characterization of *Salvia* species used in Valencian traditional herbal preparations

Vanessa Martínez-Francés, Emeline Hahn, Segundo Ríos, Diego Rivera, Eike Reich, Roser Vila, Salvador Cañigueral*

*** Correspondence:** Prof. Salvador Cañigueral: s.canigueral@ub.edu

Supplementary Table 1. Composition of the essential oils of *Salvia blancoana* subsp. *mariolensis*, *S.* x *hegelmaieri* and *S. officinalis* subsp. *lavandulifolia* from the Valencia region (Spain) and close areas.

Retention indices[1]				Constituents[2]	Percentage in the essential oils [3]											Identification methods[1,4]
A	B	C	D		SB1	SB2	SB3	SB4	SB5	SB6	SB7	SH1	SL1	SL2	SL3	
1017	922	109	207	Tricyclene	0.1	t	0.1	0.2	0.2	0.3	0.2	0.2	0.1	0.2	0.3	A,B,C,D,E
1030	933	115	211	α-Pinene	3.6	3.0	4.2	6.5	4.9	7.4	4.3	6.0	3.7	9.5	3.1	A,B,C,D,E
1030	924	115	208	α-Thujene	0.9	t	0.7	0.5	0.2	0.2	t	t	0.1	0.1	1.8	A,B,C,D,E
1066	945	131	217	α-Fenchene	t	t	t	t	t	t	t	t	t	t	t	A,B,C,D,E
1077	946	135	218	Camphene	4.2	6.0	4.7	6.0	5.6	8.5	4.6	5.8	3.8	5.6	7.6	A,B,C,D,E
1118	973	156	229	β-Pinene	5.0	7.6	5.7	6.3	6.0	5.1	5.8	5.8	3.1	6.3	7.7	A,B,C,D,E
1128	966	162	227	Sabinene	0.7	1.8	1.0	0.6	1.3	0.6	1.3	1.5	t	0.5	1.9	A,B,C,D,E
1130	948	164	219	Verbenene	-	t	t	t	-	-	t	t	t	t	0.1	A,B,C,D,E
1137	937	168	214	2,4(10)-Thujadiene	-	-	-	t	-	-	-	-	-	-	0.2	A,B,C,D,E
1151	1000	176	242	δ-3-Carene	t	t	t	t	t	t	-	-	-	t	t	A,B,C,D,E
1163	984	184	233	Myrcene	5.6	10.0	3.0	5.0	3.7	5.4	4.0	4.3	3.7	5.0	2.5	A,B,C,D,E
1170	1000	188	240	α-Phellandrene	0.1	t	t	t	t	0.1	t	t	0.1	t	t	A,B,C,D,E
1185	1011	197	246	α-Terpinene	0.2	0.2	0.1	0.1	0.2	0.2	0.2	0.2	0.3	0.4	0.4	A,B,C,D,E

Retention indices[1]				Constituents[2]	Percentage in the essential oils [3]											Identification methods[1,4]
A	B	C	D		SB1	SB2	SB3	SB4	SB5	SB6	SB7	SH1	SL1	SL2	SL3	
1193	-	201	-	3-Hexanol	-	-	t	-	-	-	-	-	t	-	-	A,C,E
1193	979	202	233	2,3-Dehydro-1,8-cineole	t	t	t	t	t	t	t	t	-	t	t	A,B,C,D,E
1205	1026	207	254	Limonene	1.8	1.2	2.1	2.7	1.7	5.0	4.5	3.3	3.3	6.7	8.2	A,B,C,D,E
1220	1026	213	254	1,8-Cineole	40.4	40.1	32.0	25.4	35.3	13.7	45.7	40.0	27.1	19.8	25.6	A,B,C,D,E
1222	-	213	-	*p*-Mentha-1,5,8-triene	-	-	t	-	-	-	t	-	t	t	t	A,C,E
1224	827	213	169	*trans*-2-Hexenal	-	t	t	-	-	-	t	t	t	t	t	A,B,C,D,E
1236	1027	221	258	*cis*-β-Ocimene	1.1	0.4	3.3	2.4	2.6	3.5	1.6	0.1	0.1	0.2	1.5	A,B,C,D,E
1252	1050	228	264	γ-Terpinene	0.6	0.5	0.7	0.5	0.4	1.3	0.5	0.8	0.6	1.0	1.8	A,B,C,D,E
1255	1038	232	263	*trans*-β-Ocimene	0.2	0.1	0.4	0.3	0.4	0.3	0.3	0.1	0.1	t	0.4	A,B,C,D,E
1261	-	238	-	3-Octanone	-	-	t	-	-	-	t	t	t	t	-	A,C,E
1280	1015	241	248	*p*-Cymene	0.5	0.1	0.8	0.9	0.3	1.0	0.8	0.4	2.0	2.5	3.3	A,B,C,D,E
1292	1080	246	276	Terpinolene	0.2	0.3	0.3	0.2	0.3	0.4	0.1	0.2	0.2	0.2	0.2	A,B,C,D,E
1309	-	255	-	Oct-1-en-3-one	-	-	t	-	-	-	-	t	-	t	-	A,C,E
1342	-	271	-	6-Methyl-5-hepten-2-one	-	-	t	t	-	-	t	t	t	t	-	A,C,E
1351	841	276	164	1-Hexanol	-	-	-	-	-	-	-	t	-	t	t	A,B,C,D,E
1374	-	288	-	3-Methyl-3-butenyl isovalerate	t	t	t	-	t	t	t	t	t	-	t	A,C,E
1377	-	290	-	allo-Ocimene	t	t	0.1	t	t	t	t	t	-	-	t	A,C,E
1365	-	283	-	Oct-1-en-3-yl acetate	t	-	t	-	t	t	-	t	t	t	-	A,C,E
1369	-	285	-	*cis*-3-Hexen-1-ol	-	-	t	-	-	-	t	t	t	-	-	A,C,E
1392	-	297	-	3-Octanol	t	t	t	t	-	-	t	t	t	t	-	A,C,E
1411	1060	308	269	Fenchone	t	-	-	-	-	t	t	-	t	-	-	A,B,C,D,E
1430	1099	317	293	Perillene	t	t	t	t	t	t	t	-	-	-	-	A,B,C,D,E
1432	1100	318	295	β-Thujone	-	-	-	-	-	-	-	-	-	t	-	A,B,C,D,E
1436	1095	320	292	α-Thujone	-	t	-	t	t	-	-	-	t	t	t	A,B,C,D,E
1448	1058	326	269	*trans*-Linalool oxide	t	0.2	t	0.3	t	t	t	-	t	t	0.4	A,B,C,D,E

Retention indices[1]				Constituents[2]	Percentage in the essential oils [3]											Identification methods[1,4]
A	B	C	D		SB1	SB2	SB3	SB4	SB5	SB6	SB7	SH1	SL1	SL2	SL3	
1450	965	326	227	Oct-1-en-3-ol	t	0.1	t	0.3	t	t	t	0.1	0.1	0.1	t	A,B,C,D,E
1454	1347	328	421	α-Cubebene	-	t	t	0.1	t	t	t	t	t	t	t	A,B,C,D,E
1454	1055	328	268	*trans*-Sabinene hydrate	0.4	0.6	0.3	0.5	0.2	0.4	0.1	0.2	0.2	0.1	0.4	A,B,C,D,E
1454	1061	328	274	Camphenilone	-	-	-	-	-	-	-	t	-	-	t	A,B,C,D,E
1458	-	330	-	*cis*-Linalool oxide	t	0.1	t	t	t	t	t	t	-	-	t	A,C,E
1479	1333	341	412	δ-Elemene	-	-	t	-	-	-	-	-	-	-	t	A,B,C,D,E
1486	1019	344	252	2-Ethyl-1-hexanol	t	t	t	t	t	-	-	t	t	t	-	A,B,C,D,E
1491	1364	347	429	α-Ylangene	-	-	-	-	-	-	-	-	0.1	0.2	t	A,B,C,D,E
1500	1219	349	353	*cis*-3-Hexenyl isovalerate	t	t	t	t	t	t	t	t	t	t	t	A,B,C,D,E
1502	1102	352	300	α-Campholenal	t	t	t	t	t	0.1	0.2	0.1	0.1	0.1	0.1	A,B,C,D,E
1510	1370	356	433	α-Copaene	t	t	t	0.1	t	t	0.1	t	0.1	0.1	t	A,B,C,D,E
1536	1126	368	304	Camphor	21.1	19.6	24.4	20.3	18.8	28.6	12.1	15.9	23.9	16.2	16.8	A,B,C,D,E
1540	1380	368	436	α-Gurjunene	-	0.1	0.1	t	0.1	t	t	t	0.1	0.1	t	A,B,C,D,E
1548	-	373	-	β-Cubebene	-	t	t	t	-	t	t	t	-	-	-	A,C,E
1551	1084	375	278	Linalool	0.5	0.3	0.9	0.2	0.4	0.5	0.3	0.3	0.2	0.2	0.6	A,B,C,D,E
1556	1084	377	278	*cis*-Sabinene hydrate	0.2	0.2	0.1	0.2	0.1	0.2	0.1	0.1	0.1	0.1	0.2	A,B,C,D,E
1566	1240	383	367	Linalyl acetate	t	-	0.3	t	0.1	0.2	0.1	t	-	t	-	A,B,C,D,E
1571	1112	384	300	*p*-Menth-2-en-1-ol	t	t	t	t	t	t	t	t	t	t	0.1	A,B,C,D,E
1574	1410	388	453	cis-α-Bergamotene	t	-	t	t	-	-	-	-	0.1	t	-	A,B,C,D,E
1585	1139	391	315	Pinocarvone	t	t	0.1	0.1	0.1	t	0.2	0.1	0.2	t	0.1	A,B,C,D,E
1585	1437	392	465	β-Gurjunene	-	-	t	-	-	-	-	-	t	0.1	-	A,B,C,D,E
1589	1212	393	352	Bornyl formate	t	0.1	t	-	t	0.1	t	t	t	-	t	A,B,C,D,E
1592	1270	395	381	Bornyl acetate	1.5	0.3	1.6	1.9	1.9	3.1	0.1	1.4	1.5	2.0	0.1	A,B,C,D,E
1593	-	396	-	Isobornyl acetate	-	t	t	-	-	-	-	-	-	-	-	A,C,E
1597	1378	397	435	β-Elemene	-	t	t	-	t	-	0.1	t	-	-	t	A,B,C,D,E

Retention indices[1]				Constituents[2]	Percentage in the essential oils [3]											Identification methods[1,4]
A	B	C	D		SB1	SB2	SB3	SB4	SB5	SB6	SB7	SH1	SL1	SL2	SL3	
1599	1432	399	463	*trans*-α-Bergamotene	-	-	-	-	-	-	-	-	t	0.2	-	A,B,C,D,E
1603	1425	401	459	*epi*-Bicyclosesquiphellandrene	-	0.1	-	t	-	t	-	t	t	t	-	A,B,C,D,E
1610	1417	403	458	β-Caryophyllene	0.2	0.7	1.5	3.5	1.2	0.4	0.4	0.4	3.6	2.4	0.6	A,B,C,D,E
1613	1163	406	323	Terpinen-4-ol	0.6	0.2	0.2	0.3	-	-	1.0	0.7	t	0.4	1.6	A,B,C,D,E
1627	1435	412	467	Aromadendrene	0.1	t	t	0.1	t	-	0.1	t	0.1	0.1	-	A,B,C,D,E
1627	-	412	-	1-Terpineol	t	t	t	-	t	t	0.1	t	t	-	-	A,C,E
1636	1172	417	332	Myrtenal	t	t	0.1	0.1	t	t	0.2	0.1	t	t	0.1	A,B,C,D,E
1655	1360	426	427	Bornyl propionate	0.1	t	0.1	0.1	0.2	0.1	0.1	t	0.1	t	0.1	A,B,C,D,E
1658	-	428	-	*allo*-Aromadendrene	-	t	t	t	t	-	-	-	t	-	t	A,C,E
1663	1216	430	354	Pulegone	-	-	t	-	-	0.1	-	t	-	0.2	-	A,B,C,D,E
1667	-	433	-	Pinocarveol	t	t	0.1	-	t	-	0.1	t	0.1	0.1	0.1	A,C,E
1675	-	437	-	Sabinyl acetate	-	-	-	1.0	0.2	-	0.2	-	t	-	-	A,C,E
1676	1142	434	323	Isoborneol	t	t	t	t	t	t	t	t	t	t	t	A,B,C,D,E
1678	1132	435	312	*cis*-Verbenol	t	t	t	t	t	t	t	-	-	-	-	A,B,C,D,E
1679	1153	436	318	δ-Terpineol	t	0.1	t	0.1	0.1	0.1	0.8	-	0.2	t	0.1	A,B,C,D,E
1679	1450	437	474	α-Humulene	t	t	t	0.5	0.1	0.6	0.2	t	1.4	0.6	0.3	A,B,C,D,E
1680	1466	438	481	γ-Selinene	-	-	-	-	-	-	-	-	-	t	0.1	A,B,C,D,E
1680	-	439	-	*trans*-*p*-Mentha-2,8-dien-1-ol	-	-	t	t	-	t	t	-	0.1	-	-	A,C,E
1681	1176	439	329	Estragole	-	t	-	-	-	-	-	0.8	0.8	2.2	-	A,B,C,D,E
1682	1131	439	308	*trans*-Verbenol	0.2	t	0.1	0.3	0.1	0.1	0.1	t	0.1	t	t	A,B,C,D,E
1684	1216	440	351	Neral	t	t	t	t	0.1	-	t	1.0	0.3	t	t	A,B,C,D,E
1684	1161	440	321	p-Mentha-1,8-dien-4-ol	t	-	t	-	-	t	-	t	t	t	t	A,B,C,D,E
1688	1472	442	484	α-Amorphene	-	t	t	0.1	-	0.1	-	t	0.3	0.2	t	A,B,C,D,E
1707	1174	451	329	α-Terpineol	0.7	0.6	0.7	0.1	0.4	0.1	1.3	0.9	1.0	0.8	1.5	A,B,C,D,E
1708	1332	451	417	α-Terpenyl acetate	0.6	-	0.2	0.6	2.3	t	-	0.1	t	0.1	t	A,B,C,D,E

Retention indices[1]				Constituents[2]	Percentage in the essential oils [3]											Identification methods[1,4]
A	B	C	D		SB1	SB2	SB3	SB4	SB5	SB6	SB7	SH1	SL1	SL2	SL3	
1713	1153	454	318	Borneol	3.0	0.8	3.7	5.2	5.1	7.4	2.7	3.4	4.6	5.1	4.2	A,B,C,D,E
1722	1343	455	418	Neryl acetate	t	-	-	-	t	t	-	-	t	-	0.1	A,B,C,D,E
1723	-	458	-	Germacrene D	t	0.1	0.2	0.1	t	0.2	t	t	-	t	-	A,C,E
1728	1500	460	496	β-Cadinene	t	-	t	-	-	-	-	-	-	0.1	0.1	A,B,C,D,E
1734	-	464	-	β-Selinene	-	-	t	t	-	-	-	t	0.1	0.2	-	A,C,E
1734	1492	464	494	α-Muurolene	-	t	0.1	-	t	t	t	t	0.1	0.1	t	A,B,C,D,E
1737	1244	466	366	Geranial	-	-	-	-	t	-	t	t	0.2	t	0.1	A,B,C,D,E
1737	1490	467	492	α-Selinene	-	-	t	-	-	-	-	-	0.2	0.1	-	A,B,C,D,E
1741	1228	469	359	Piperitone	-	-	t	-	-	-	0.1	t	-	-	t	A,B,C,D,E
1744	1217	470	354	Carvone	-	-	t	-	-	-	-	t	t	t	0.2	A,B,C,D,E
1746	-	471	-	α-Zingiberene	-	-	t	-	-	-	-	-	t	t	-	A,C,E
1748	1485	471	490	Bicyclogermacrene	0.2	0.2	0.2	0.1	0.3	0.1	0.2	0.1	-	-	t	A,B,C,D,E
1749	1348	472	422	α-Longipinene	-	-	-	t	-	-	-	-	-	-	-	A,B,C,D,E
1749	1189	472	340	*cis*-Piperitol	-	t	t	t	-	-	t	-	-	-	t	A,B,C,D,E
1751	1193	473	342	*trans*-Piperitol	-	-	t	-	-	0.1	t	t	t	t	t	A,B,C,D,E
1763	1360	478	427	Geranyl acetate	t	t	t	-	t	0.1	-	t	0.2	0.1	t	A,B,C,D,E
1768	1513	480	504	δ-Cadinene	0.1	0.4	0.2	0.3	0.3	0.2	0.1	0.4	0.4	0.3	0.1	A,B,C,D,E
1771	1505	482	500	γ-Cadinene	-	t	t	0.2	t	0.2	t	0.1	0.2	0.1	t	A,B,C,D,E
1784	1469	488	482	*ar*-Curcumene	0.1	0.1	0.1	0.5	-	-	-	t	0.4	0.1	-	A,B,C,D,E
1791	1537	492	513	Selina-3,7(11)-diene	-	-	-	-	-	-	-	t	0.1	0.1	-	A,B,C,D,E
1793	1522	492	508	Cadina-1,4-diene	-	t	t	t	-	-	-	t	t	-	t	A,B,C,D,E
1794	1202	492	344	Cumin aldehyde	-	-	-	-	-	-	t	t	t	t	t	A,B,C,D,E
1794	1434	492	463	Neryl propionate	-	-	t	-	-	-	-	t	0.1	-	t	A,B,C,D,E
1796	1131	494	311	*cis*-Sabinol	-	-	-	-	-	-	-	-	-	t	0.1	A,B,C,D,E
1803	1180	496	334	Myrtenol	t	t	0.1	0.2	t	0.1	0.2	0.1	0.1	0.1	0.2	A,B,C,D,E

Retention indices[1]				Constituents[2]	Percentage in the essential oils [3]											Identification methods[1,4]
A	B	C	D		SB1	SB2	SB3	SB4	SB5	SB6	SB7	SH1	SL1	SL2	SL3	
1807	1202	498	344	Nerol	t	t	0.1	-	t	t	t	t	0.1	t	0.1	A,B,C,D,E
1817	1489	504	495	Geranyl isobutyrate	0.2	t	t	-	t	0.1	0.1	0.1	0.1	t	t	A,B,C,D,E
1826	1451	507	471	Geranyl propionate	0.9	0.3	0.6	0.1	-	0.4	t	0.4	0.2	0.1	0.1	A,B,C,D,E
1843	1260	516	376	*trans*-Anethole	-	-	-	-	-	-	-	t	-	t	-	A,B,C,D,E
1844	1551	517	520	Germacrene B	-	-	t	-	-	-	-	-	-	-	t	A,B,C,D,E
1845	1190	516	339	*trans*-Carveol	-	t	t	0.1	0.2	0.1	0.1	t	t	t	0.1	A,B,C,D,E
1845	1509	516	502	*cis*-Calamenene	-	t	-	-	-	-	t	t	0.2	0.2	-	A,B,C,D,E
1853	1236	520	363	Geraniol	0.3	0.1	0.3	0.2	0.2	0.3	0.4	0.3	0.8	0.3	0.6	A,B,C,D,E
1856	-	525	-	Thymyl acetate	-	-	-	-	-	-	-	-	t	-	-	A,C,E
1860	-	524	-	*p*-Cymen-8-ol	-	-	t	t	-	t	0.1	t	-	-	0.1	A,C,E
1867	-	527	-	Geranyl acetone	-	-	t	-	-	-	-	t	-	-	-	A,C,E
1875	1202	537	344	*cis*-Carveol	-	-	t	t	-	-	t	t	t	-	0.1	A,B,C,D,E
1900	1556	543	515	Geranyl butyrate	-	-	0.1	-	-	-	-	t	0.1	0.1	t	A,B,C,D,E
1901	-	546	-	Neryl isovalerate	-	-	t	-	-	-	-	-	t	-	-	A,C,E
1902	1594	545	542	Geranyl isovalerate	0.2	-	t	-	0.2	-	-	t	0.6	0.1	t	A,B,C,D,E
1924	1528	555	511	α-Calacorene	-	-	t	-	-	-	t	t	0.3	0.3	t	A,B,C,D,E
1936	1310	560	400	Piperitenone	-	-	t	-	-	-	-	t	-	t	t	A,B,C,D,E
1944	1560	564	524	Palustrol	t	t	t	t	0.1	-	t	t	-	-	t	A,B,C,D,E
1947	-	566	-	Shyobunol	-	-	t	t	t	-	-	t	-	-	t	A,C,E
1951	1369	567	432	*cis*-Jasmone	t	-	t	-	-	-	-	t	t	t	-	A,B,C,D,E
1999	1565	590	528	*endo*-1-Bourbonanol	-	-	-	-	-	-	-	t	-	0.06	-	A,B,C,D,E
2003	1570	593	531	Caryophyllene oxide	0.6	0.3	0.9	1.2	0.8	0.8	0.8	0.4	1.9	1.0	1.5	A,B,C,D,E
2015	1279	599	385	Perilla alcohol	-	-	-	-	-	-	t	t	-	-	t	A,B,C,D,E
2023	1369	603	431	Methyl eugenol	-	-	-	-	-	-	-	0.3	t	0.1	t	A,B,C,D,E
2037	-	610	-	Ledol	-	-	t	t	t	t	t	t	-	0.1	t	A,C,E

Martínez-Francés et al. (2017) *Front. Pharmacol.* 8:467.
doi: 10.3389/fphar.2017.00467

Retention indices[1]				Constituents[2]	Percentage in the essential oils [3]											Identification methods[1,4]
A	B	C	D		SB1	SB2	SB3	SB4	SB5	SB6	SB7	SH1	SL1	SL2	SL3	
2042	-	613	-	Nerolidol	-	t	t	-	-	-	-	t	-	t	-	A,C,E
2049	1598	616	544	Humulene oxide	-	-	t	-	-	0.3	t	0.1	0.6	0.1	0.2	A,B,C,D,E
2060	1564	622	528	1,6-Germacradien-5-ol	0.1	0.1	0.3	t	0.2	0.1	0.3	0.1	t	t	0.1	A,B,C,D,E
2070	-	627	-	10-*epi*-Cubenol	-	-	t	t	t	t	0.1	t	t	t	t	A,C,E
2077	-	629	-	1-*epi*-Cubenol	-	-	t	t	t	t	t	t	-	t	t	A,C,E
2072	-	637	-	Globulol	-	t	t	t	t	-	t	t	-	t	t	A,C,E
2097	1582	641	537	Viridiflorol	-	t	t	t	0.4	t	t	t	1.8	3.7	0.1	A,B,C,D,E
2140	1564	662	528	Spathulenol	0.9	0.3	0.3	1.3	0.5	0.2	1.0	0.5	0.1	0.1	0.1	A,B,C,D,E
2180	1329	679	415	Eugenol	-	-	t	-	-	t	-	t	t	t	t	A,B,C,D,E
2183	-	681	-	Bisabolol oxide B	-	-	-	-	-	-	0.1	-	-	-	-	A,C,E
2184	1620	682	557	T-Cadinol	0.1	0.2	0.1	0.1	t	-	t	0.1	0.2	0.2	0.1	A,B,C,D,E
2199	1639	689	564	T-Muurolol	-	-	t	-	0.1	-	t	0.2	t	0.1	t	A,B,C,D,E
2218	1610	693	554	δ-Cadinol	-	-	t	t	t	-	0.1	t	0.2	t	t	A,B,C,D,E
2228	1267	695	377	Thymol	-	-	-	-	-	-	-	t	0.1	0.1	0.1	A,B,C,D,E
2236	-	705	-	α-Eudesmol	-	-	-	-	-	-	-	-	t	0.10	-	A,C,E
2243	1280	699	394	Carvacrol	-	-	t	-	-	-	-	t	0.1	0.1	0.1	A,B,C,D,E
2243	-	699	-	α-Bisabolol	-	-	-	-	-	-	-	t	-	-	-	A,C,E
2245	1633	711	561	β-Eudesmol	-	-	t	-	-	-	-	-	0.4	0.2	t	A,B,C,D,E
2266	1612	706	550	Isospathulenol	-	-	t	t	t	-	t	t	0.1	0.1	0.1	A,B,C,D,E
2279	-	710	-	Elemicin	-	-	-	-	-	-	-	0.3	-	-	-	A,C,E
2279	1639	710	564	α-Cadinol	0.1	0.2	0.3	0.1	0.1	0.2	0.1	0.5	0.1	t	0.2	A,B,C,D,E
>2300	1653	758	570	Cinnamyl isovalerate	-	-	t	-	-	-	-	-	t	-	t	A,B,C,D,E
-	1141	-	316	Camphene hydrate[5]	t	-	-	t	-	t	-	-	-	t	0.1	B,D,E
-	1175	-	336	β-Fenchyl alcohol[5]	t	-	t	-	t	0.6	-	0.1	-	-	-	B,D,E
-	1230	-	360	*trans*-Cinnamaldehyde[5]	-	-	-	-	-	-	-	-	-	-	t	B,D,E

Retention indices[1]				Constituents[2]	Percentage in the essential oils [3]											Identification methods[1,4]
A	B	C	D		SB1	SB2	SB3	SB4	SB5	SB6	SB7	SH1	SL1	SL2	SL3	
-	1437	-	465	α-Guaiene[5]	-	-	-	-	-	-	-	-	-	t	-	B,D,E
-	1480	-	489	Valencene[5]	-	-	t	-	-	-	-	-	0.1	t	-	B,D,E
-	1481	-	487	γ-Muurolene[5]	-	-	-	-	-	t	-	-	-	t	-	B,D,E
-	1482	-	490	δ-Selinene[5]	-	-	t	t	-	-	-	-	t	-	-	B,D,E
-	1492	-	495	Neryl butyrate[5]	-	-	-	-	-	-	-	-	t	-	-	B,D,E
-	1501	-	497	β-Cadinene[5]	-	-	-	-	-	-	-	-	t	-	-	B,D,E
-	1521	-	499	α-Elemene[5]	-	-	-	-	-	-	-	t	-	-	t	B,D,E
-	1529	-	510	β-Calacorene[5]	-	-	-	-	-	-	-	-	-	t	-	B,D,E
-	1541	-	511	*trans*-Sesquisabinene hydrate[5]	-	-	-	-	-	-	-	t	-	0.1	-	B,D,E
-	1610	-	554	Fonenol[5]	-	-	-	-	-	-	-	-	0.1	-	-	B,D,E
-	1674	-	575	Juniper camphor[5]	-	-	-	-	-	-	-	-	0.4	-	-	B,D,E
**Composition by groups of constituents**																
				Monoterpene hydrocarbons	24.7	31.3	27.3	32.3	27.7	39.3	28.3	28.8	21.3	38.2	41.1	
				Oxygenated monoterpenes	71.2	63.8	66.3	57.5	66.2	56.8	66.7	65.6	62.6	46.5	54.4	
				Sesquiterpene hydrocarbons	0.8	1.8	2.6	5.7	2.1	1.9	1.3	1.1	8.1	5.7	1.4	
				Oxygenated sesquiterpenes	1.8	1.1	2.0	2.8	2.3	1.6	2.6	2.0	5.8	5.9	2.5	
				Phenylpropanoids	-	t	t	-	-	t	-	1.4	0.8	2.3	t	
				Others	0.1	0.2	0.1	0.3	t	t	0.1	0.2	0.2	0.2	t	
				**Total identified**	98.5	98.2	98.3	98.5	98.3	99.6	98.9	99.1	98.9	98.8	99.3	

1) Retention indices: A: n-Alkane indices in Supelcowax™-10; B: n-Alkane indices in Equity-1™; C: FAME indices in Supelcowax™-10; D: FAME indices in Equity-1™.
2) Compounds listed by elution order in the polar column (Supelcowax™-10) except the last fourteen constituents.
3) For the meaning of the identification codes, see Table 1. t: traces (≤ 0.05%).
4) Identification method E: GC-MS.
5) Constituents only detected in the Equity-1™ column.
